# Behaviors of Micro-Arcs, Bubbles, and Coating Growth during Plasma Electrolytic Oxidation of β-Titanium Alloy

**DOI:** 10.3390/ma16010360

**Published:** 2022-12-30

**Authors:** Toshiaki Yasui, Katsuki Hayashi, Masahiro Fukumoto

**Affiliations:** Department of Mechanical Engineering, Toyohashi University of Technology, Toyohashi 441-8580, Japan

**Keywords:** plasma electrolytic oxidation, PEO, micro-arc, β-titanium alloy, discharge crater, discharge pore

## Abstract

The plasma electrolytic oxidation (PEO) of a titanium alloy, Ti-15V-3Cr-3Sn-3Al, was performed to develop mechanical applications by improving the tribological characteristics. The behaviors of micro-arcs, bubbles, and coating growth during the PEO process were investigated under three different operating conditions, constant voltage (CV) operation, constant current operation (CC), and short treatment time (ST) operation, to control the surface structure and function by the PEO process. A low friction coefficient was achieved by CV operation at 500 V and by CC operation at 3.0 kA/m^2^. The maximum coating thickness of 6.88 μm was achieved by CV operation at 500 V and 60 s. From the observation of micro-arcs, bubbles, and discharge craters by ST operation, the minimum discharge diameter of the micro-arc was 8 μm, and the discharge craters had a discharge pore size of 0.3 μm in diameter in the center with a petal-shaped burr around the discharge pore. During the PEO process, no bubble bursts around the micro-arcs and no backfilling of the discharge pores by the ejected materials were observed. Thus, the discharge pores remain a porous structure in the PEO coating for Ti. The utilization efficiency of the total charge density by CV operation above 300 V was lower than that by the conventional anodization process. The utilization efficiency of total charge density by CC operation was higher than that by the conventional anodization process.

## 1. Introduction

**Figure 1:**
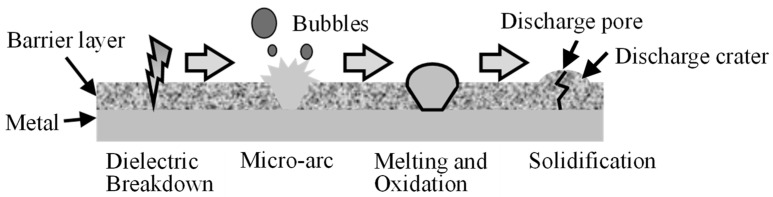
Formation process of PEO coating.

Plasma electrolytic oxidation (PEO) is a type of anodizing process with the accompanying generation of numerous small and minute spark discharges (micro-arcs) on the anode surface. The process fabricates compact, thick, and uniform oxide coatings on light metals, such as aluminum, titanium, and magnesium. This process greatly improves the wear and corrosion resistance of the light metals and has attracted attention in various industries [1,2,3,4,5]. However, the fabricated coating structures, compositions, and functional properties differ according to the metals, electrolyte components, and electrical conditions (applied voltage, input current, and waveform). Active research was conducted to improve the process and properties and elucidate the coating formation mechanism [2,3,4,5,6,7]. Figure 1 shows the formation process of a PEO coating. Individual micro-arcs locally melt and eject the anode material from the surface with accompanying gas evolution as bubbles at the surrounding. After extinction of the micro-arcs, the ejected material is cooled, condensed, and solidified on the anode and forms a discharge crater on the anode. The ejection point on the anode remains as a discharge pore in the center of the discharge crater. The behavior of micro-arcs and bubbles can be attributed to the processing conditions. These micro-arcs and bubbles influence the structures, the composition, and the functional properties of the coating. Thus, observation of micro-arcs during the PEO process has attracted considerable attention for optimizing the process and coating properties [7,8,9,10,11,12,13,14,15]. However, there are no reports on the bubbles formed during the PEO process.

PEO of titanium and its alloys has been widely used in industry. Although Ti and its alloys have high specific strength, high corrosion resistance, and biocompatibility, they have high chemical reactivity and poor tribological properties. For biomedical applications such as dental and orthopedic implants, it is necessary to enhance the bonding strength and bioactivity between the bio-tissue and the implant. Porous and bio-active coatings can be fabricated on a Ti implant surface by a PEO process [2,3,4,16,17]. As for mechanical applications, such as aerospace and automotive industries, the PEO process improved the surface properties of Ti alloys for corrosion resistance, wear resistance, and thermal control [2,18,19,20,21]. 

The surface structures of PEO coating for Ti and its alloys were porous and very different from those of Al [7]. In addition, the properties differed according to the type of titanium alloy [22]. The differences were attributed to the difference in generated micro-arcs and bubbles during the PEO process. There are some reports on the effects of the kinds of alloy and process conditions for the β-titanium alloy, Ti-15V-3Cr-3Sn-3Al (Ti-15-3), which is useful for mechanical applications by improving the tribological characteristics by PEO [16,22,23,24]. However, there are few reports on the effect of micro-arcs and bubbles on the PEO process of Ti and its alloys. The behavior of micro-arcs and bubbles controls the surface structure and the function of the PEO coating. Therefore, it is necessary to clarify the effects of the applied voltage and input current between the electrodes on the PEO process for controlling the behavior of micro-arcs and bubbles.

This study investigated the behaviors of micro-arcs, bubbles, and coating growth using the PEO process for the β-titanium alloy to understand the coating formation process. The behaviors of micro-arcs and bubbles during the PEO process and the fabrication of discharge craters were observed after a short treatment time operation. The growth behaviors of the coating by constant voltage operation and constant current operation were evaluated by observing the surface and cross-section morphology and friction test. The effect of micro-arcs and bubbles on coating formation were assessed by evaluating the utilization efficiency of the total charge density.

## 2. Materials and Methods

A rectangular Ti-15-3 plate, 1.6 mm in thickness, was used as a specimen. The specimen surface was partially masked with fluorine resin tape to make a rectangular treatment area (50 mm^2^) for the PEO process. The specimen was used as the anode, and a rectangle SUS410 plate was used as the cathode. Both electrodes were immersed in the electrolyte solution within a glass container and connected to a DC power supply (Takasago, Kawasaki, Japan, HV1.0-5). The DC power supply was regulated by voltage constant (max 1 kV) mode or current constant (max 0.5 A) mode. The electrolyte containing 0.1 mol/L H_2_PO_4_ and 0.1 mol/L H_2_SO_4_ were prepared from reagent chemicals and purified water. 

**Figure 2:**
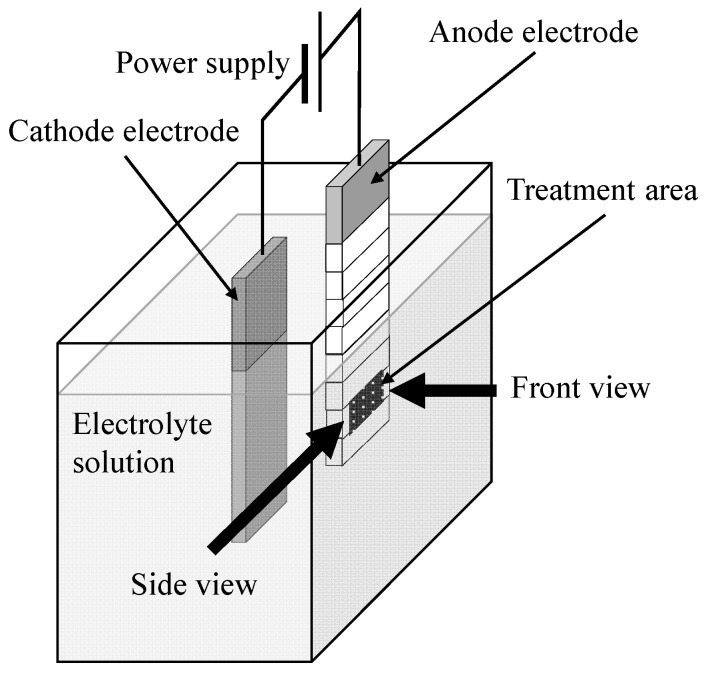
Experimental apparatus of the PEO process for the Ti-15-3 and observation direction of micro-arcs, ejected materials, bubbles, and coating surface during the PEO process.

Figure 2 presents the experimental apparatus of the PEO process for Ti-15-3 and the observation direction of micro-arcs, ejected materials, and bubbles during the PEO process. The behaviors of the micro-arcs on the anode were observed from the front of the anode (front view) using a high-speed camera (Photron, Tokyo, Japan, FASTCAM Mini AX200) at a frame speed of 100,000 fps and a 10 μs exposure time with a micro-lens (Nikon, Tokyo, Japan, Micro-Nikkor 200 mm). The behaviors of the micro-arcs, ejected materials, and bubbles on the anode were observed from the side edge of the anode (side view) using a high-speed camera at a frame speed of 25,000 fps and a 10 μs exposure time.

**Figure 3:**
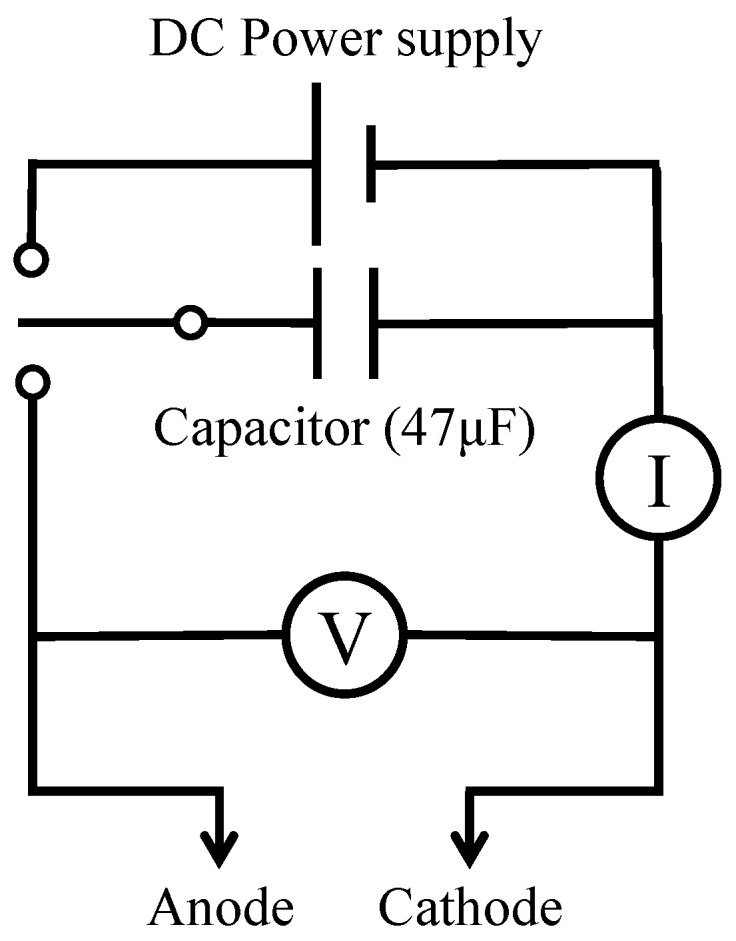
Power supply for short treatment time operation.

The effects of the applied voltage and current between the electrodes on micro-arcs and coating were investigated with three patterns of operating conditions, as shown in Table 1. In constant voltage (CV) operation, the applied voltage between the electrodes was set to 100–500 V during the during the PEO process. In constant current (CC) operation, the current density between the electrodes was set to a constant current density of 1.0 kA/m^2^ (50 mA of input current) or 3.0 kA/m^2^ (150 mA of input current) during the PEO process. In a short treatment time (ST) operation, the discharge current was supplied from a capacitor (47 μF) at a charging voltage of 500 V, as shown in Figure 3, and a short treatment time of <0.1 s was achieved at a 1 mm^2^ treatment area. This enables observations of an individual discharge crater for the understanding of the formation mechanism of the coating. The behaviors of the micro-arcs, ejected materials, and bubbles were observed for ST operation. The supplied voltage and current were measured using a data logger (Graphtec, Yokohama, Japan, midi LOGGER GL200A) and an oscilloscope (Tektronix, Beaverton, OR, USA, TDS2014B). All the experiments did not control the electrolyte temperature during the process. Thus, the evaluation of the coating was conducted at 60 s of treatment time to minimize the effect of the electrolyte temperature rise on the coating.

The surface and cross-section morphologies of the coating were observed by scanning electron microscopy (SEM, JEOL, Tokyo, Japan, JSM-6390TY), field emission SEM (FESEM, Hitachi, Tokyo, Japan, SU8000), and laser microscopy (OLYMPUS, Tokyo, Japan, OLS31-SU). For cross-section observation of the coating, an embedding resin was used to protect the coating during cutting. The coating thickness was not uniform on the anode surface. Thus, the average coating thickness, which was calculated by dividing the observed cross-section area of the coating by the observed coating width, was evaluated. The surface roughness of the specimen and coating was evaluated via laser microscopy.

**Figure 4:**
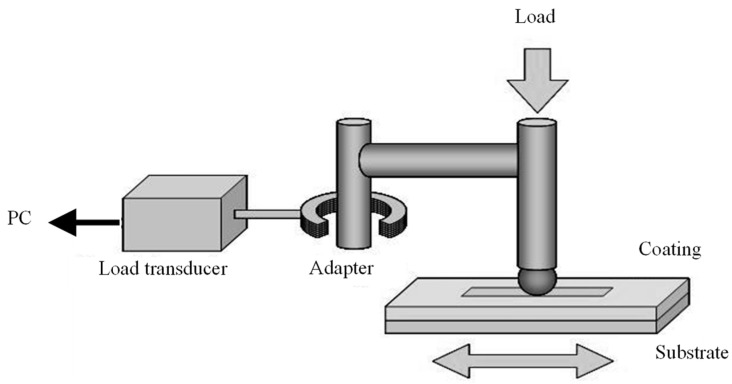
Experimental setup for the friction test.

The tribological behavior of the coating surface was evaluated using a friction tester (SHINTO Scientific, Tokyo, Japan, TYPE:14FW), as shown in Figure 4. The counterpart was a 3 mm diameter steel ball bearing (SUJ2). The tests were repeated 30 times with and without a lubricant (Synthetic oil 15W-50) under normal loads of 0.98 N for a friction speed of 5 mm/s with a friction distance of 5 mm at ambient condition of temperature and humidity. The friction coefficient was evaluated with the tester.

## 3. Results and Discussion

### 3.1. PEO Process and Coating by CV and CC Operation

**Figure 5:**
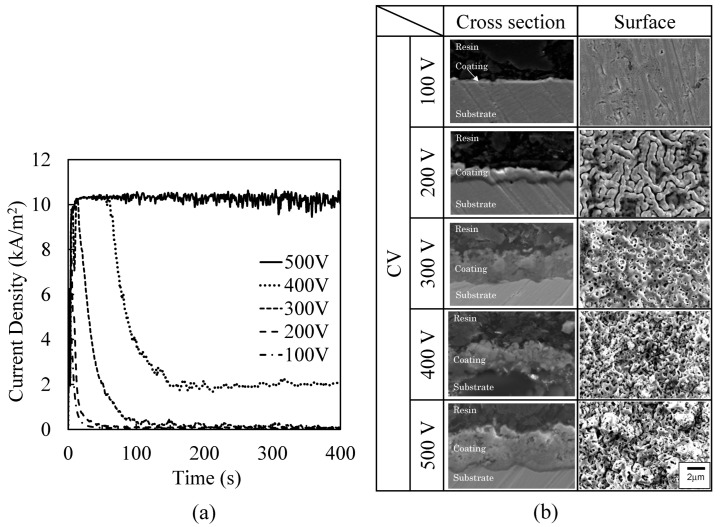
Current density and PEO coating by CV operation. (**a**) Time variation of current densities for a 400 s treatment time. (**b**) Cross-section and surface morphology of the PEO coating at a 60 s treatment time.

Figure 5a shows the time variation of current densities between the electrodes during PEO process by CV operation for a 400 s treatment time. When the applied voltage was ≤300 V, the current densities reached the maximum value (4.52 kA/m^2^ at 2 s for 100 V, 7.83 kA/m^2^ at 4 s for 200 V, 10.2 kA/m^2^ at 14 s for 300 V) and decreased to zero with time. When the applied voltage was ≥400 V, the current density at the maximum current density was 10.6 kA/m^2^, which corresponds to the maximum available current from the power supply. The current density decreased to approximately 2.0 kA/m^2^ for 400 V but held at the maximum current density for 500 V. The visible light emission of micro-arcs on the coating surface was extinguished at 21 s at 100 V, 26 s at 200 V, 51 s at 300 V, 61 s at 400 V, and 84 s at 500 V. Therefore, it is necessary to apply a voltage higher than the dielectric breakdown voltage of the coating to sustain micro-arcs. Thus, the coating growth decreased to zero due to a decrease in current density by CV operation. However, the current density at 400 V or higher did not decrease to zero with time after the micro-arcs were extinguished. This is thought to be due to the difference of the resistivity of the PEO coating.

Figure 5b shows the cross-section and the surface morphology of the PEO coating with different applied voltages by CV operation at 60 s of treatment time. The coating thickness increased with the applied voltage from 2.47 μm at 200 V to 6.88 μm at 500 V, as shown in Table 2. Conversely, the discharge craters on the surface became smaller as the applied voltage was increased. The surface morphology at 200 V shows that numerous discharge craters without discharge pores formed short strings with small gaps between the strings. The surface morphology at 500 V showed that the discharge craters with discharge pores joined with neighboring discharge craters. 

The resistivity of the PEO coating is evaluated from the applied voltage, the current density at 400 s, the treatment area, and the coating thickness, as shown in Table 2. The resistivities of the PEO coating not more than 300 V are higher than that of rutile TiO_2_ at 773 K, as shown in Table 3 [25]. The resistivities of the PEO coating at 400 V or higher are lower than that of rutile TiO_2_ at 773 K. Thus, the current density did not decrease to zero with time after micro-arc extinguishment at 400 V or higher. This can be attributed to the effect of the temperature rise on the PEO coating by micro-arc generation in the PEO coating. Although the visible light emission of micro-arcs from the PEO coating surface were extinguished, there is a possibility of micro-arc generation inside of the pore in the PEO coating [7]. This micro-arc generation in the PEO coating will change the surface structure of the coating at 400 V or higher.

**Figure 6:**
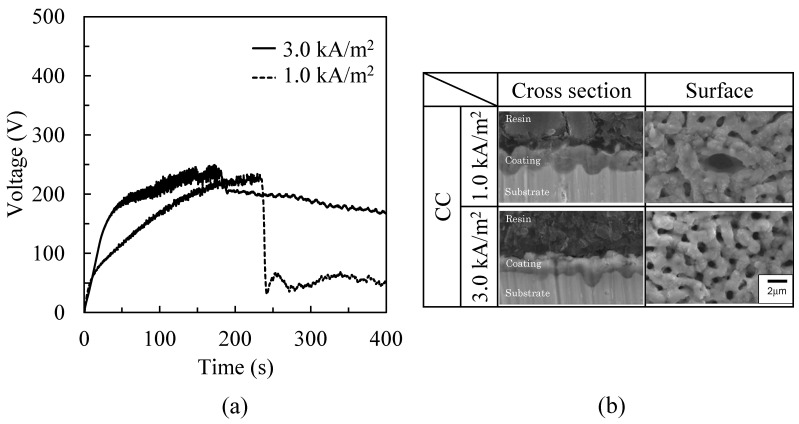
Voltage and PEO coating by CC operation. (**a**) Time variation of the voltages for a 400 s treatment time. (**b**) Cross-section and surface morphology of a PEO coating at a 60 s treatment time.

Figure 6a shows the time variation of voltage between the electrodes during the PEO process by CC operation for a 400 s treatment time. The voltage increased gradually with time until the maximum voltage of 225.7 ± 9.2 V for 1.0 kA/m^2^ and 236.8 ± 12.6 V for 3.0 kA/m^2^. After the maximum voltage, the voltage decreased to approximately 53.3 ± 0.5 V for 1.0 kA/m^2^ and approximately 167.5 ± 0.7 V for 3.0 kA/m^2^. The visible light emission of micro-arcs on the coating surface extinguished at 241 s at 1.0 kA/m^2^ and 179 s at 3.0 kA/m^2^. These observations coincided with the start time of the decreasing voltage. 

Figure 6b shows the cross-section and surface morphology of the PEO coating with different input current densities by CC operation at 60 s of treatment time. There was no obvious change in morphology and thickness of the coating, as shown in Table 4. Although the discharge craters join with neighboring craters, each discharge crater could be distinguished. The size of each discharge crater and discharge pore by CC operation was larger than that by CV operation, as shown in Figure 5b. 

The resistivity of the PEO coating by CC operation was evaluated, as shown in Table 4. The resistivity of the PEO coating for 1.0 and 3.0 kA/m^2^ are almost the same value and higher than that of rutile TiO_2_ at 773 K, as shown in Table 3. Thus, the voltage difference between 1.0 and 3.0 kA/m^2^ after micro-arc extinguishment is attributed to the difference in the input current density. These differ with the case of CV operation and lead to the difference of surface structure of the PEO coating between CV operation and CC operation.

### 3.2. Surface Properties of PEO Coating

Table 5 lists the surface roughness and friction coefficient of the coatings by friction test. The surface roughness increased with the applied voltage by CV operation. However, the friction coefficient decreased as the applied voltage was increased. The roughness and friction coefficients were low and decreased as the input current density was increased by CC operation. In comparison with non-coated materials, the lubricated PEO coating is effective for reduction of friction coefficient by the porous structure. Thus, coating with a low friction coefficient was achieved by CV operation of 500 V and by CC operation of 3.0 kA/m^2^. This is thought to be because of the densification of the PEO coating. However, the reason is not clear.

### 3.3. Micro-Arcs’ Behavior during the PEO Process

**Figure 7:**
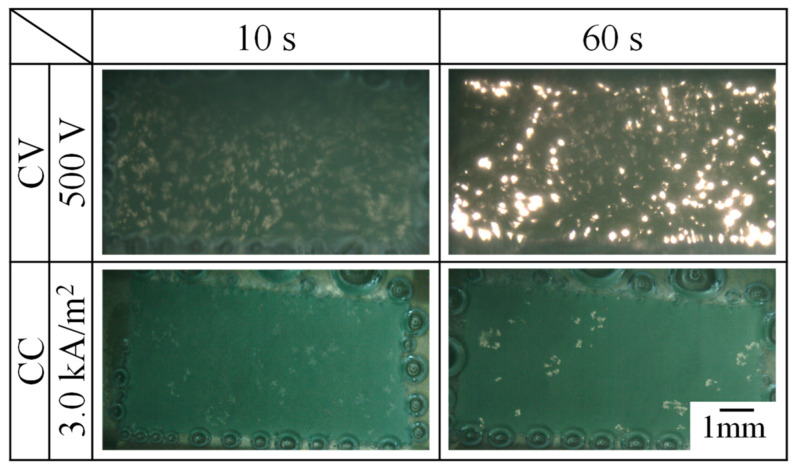
Behavior of micro-arcs in the front view during the PEO process by CV operation and by CC operation.

Figure 7 shows the behavior of micro-arcs in the front view (Figure 2) on the treatment area at 10 s and 60 s after the PEO process was started by CV operation of 500 V and by CC operation of 3.0 kA/m^2^. The micro-arcs were observed as small white traces within the rectangular treatment area. The evolved gas as bubbles surrounded the treatment area and could not be identified within the generation area of micro-arcs during the PEO process in the front view. For the PEO process by CV operation, micro-arcs were distributed widely over the surface, and the emission intensity became stronger with time. This behavior coincided with the time variation of the current density during the PEO process by CV operation in Figure 5a. For the PEO process by CC operation, micro-arcs were sparsely distributed over the surface, and the area increased with time. This behavior coincided with the time variation of the voltage during the PEO process by CC operation shown in Figure 6a.

**Figure 8:**
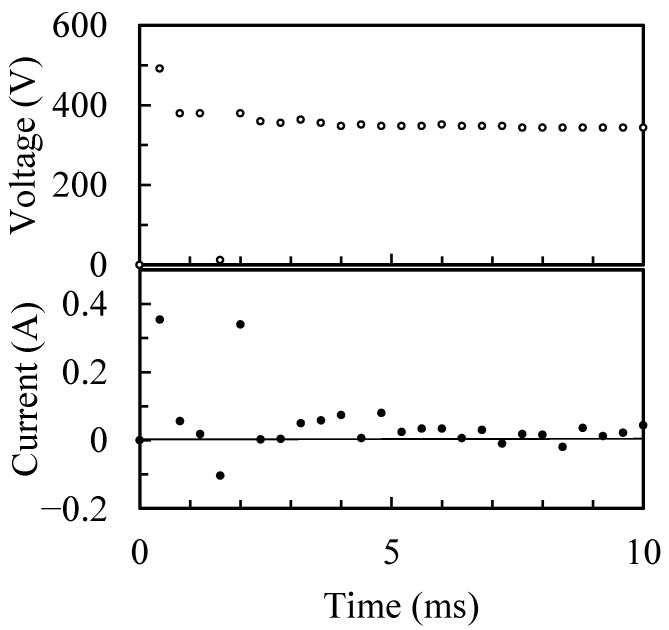
Time variation of voltage and current during the PEO process by ST operation.

**Figure 9:**
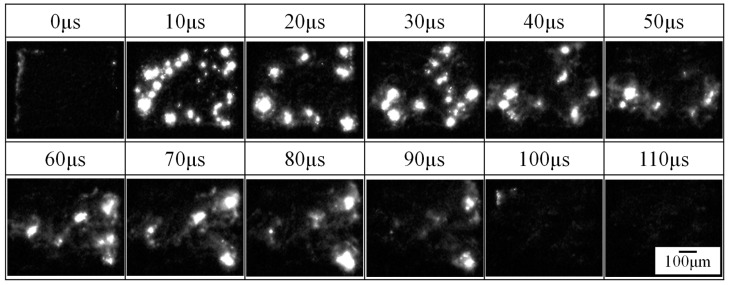
Behavior of micro-arcs in the front view during the PEO process by ST operation.

The behaviors of the individual micro-arcs during the PEO process and individual discharge craters after the process were observed by ST operation. Figure 8 presents the time variation of voltage and current during the PEO process by ST operation for 1 mm^2^ of treatment area. The ST operation enables the observation of individual discharge craters fabricated by individual micro-arcs at the beginning of the PEO process. In this experiment, the time resolution of voltage and current was 0.4 ms, and it was difficult to evaluate the discharge pulse exactly. However, the first discharge pulse was recorded at less than 1 ms after starting of the PEO process by ST operation. The recorded peak current of the first pulse was 0.354 A as the minimum peak current during the ST operation. Thus, the minimum time-averaged current during 10 ms of ST operation was calculated as 47.4 mA, which corresponds to 47.4 kA/m^2^ of current density, which is 4.5 times higher than that of CV operation of a 500 V applied voltage, as shown in Figure 5a. Figure 9 shows the behavior of micro-arcs in the front view (Figure 2) after starting of the PEO process by ST operation within 110 μs. Small and minute spark discharges (micro-arcs) were generated on the anode. Individual micro-arcs have a diameter ranging from approximately 8 to 100 μm and discharge times of <40 μs. However, the discharge crater was less than a few μm on the coating surface, as shown in Figure 5b and Figure 6b. The micro-arc with a large discharge diameter is believed to be the combined discharge with small micro-arcs or a cascade of discharges [14]. This result was attributed to the limitation of space–time resolution for the observation system. Thus, the estimated diameter of the individual micro-arcs at the minimum value is 8 μm.

**Figure 10:**
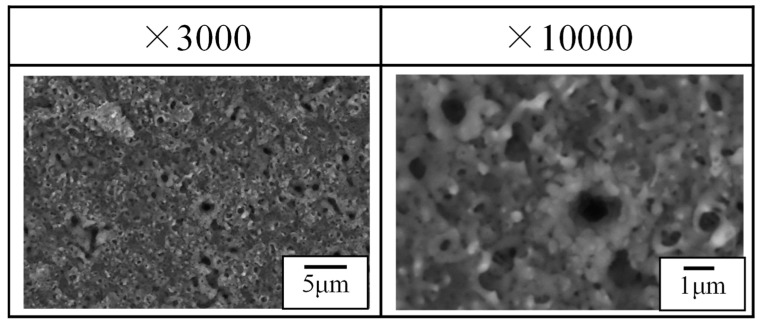
Discharge craters on the anode surface after the PEO process by ST operation.

Figure 10 shows the individual discharge craters on the anode surface after the PEO process by ST operation. The individual discharge craters have a discharge pore diameter of 0.3 μm in diameter in the center and a petal-shaped burr diameter of a few μm in diameter around the discharge pore. From the observation of micro-arcs in Figure 9, the size of the discharge craters was smaller than the estimated discharge diameter of micro-arcs (8 μm). The burr was fabricated by the ejected material through the discharge pore on the center of the discharge crater. However, the surface structure differs with the PEO coating, as shown in Figure 5b and Figure 6b. This was attributed to the subsequent discharges during the PEO process. The petal-shaped burr melted and joined with neighboring craters by the discharge. The discharge pores remained on the surface and fabricated a porous structure in the coating when there was no backfilling process.

**Figure 11:**
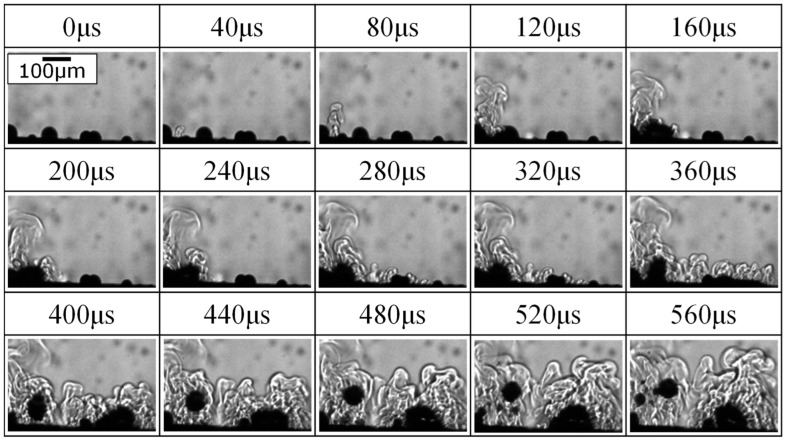
Behavior of micro-arcs, ejected materials, and bubbles in the side view during the PEO process by ST operation.

Figure 11 presents the behavior of micro-arcs, ejected materials, and bubbles in the side view (Figure 1) after starting of PEO process by ST operation within 560 μs. Many floating bubbles in the solution were observed as black blurred spots on the far side of the focal plane. Several bubbles ranging from 25 to 62 μm in diameter were observed on the anode surface as a black hemisphere in the focal plane at 0 μs. A small fringe pattern was observed at 40 μs, and a small light emission (micro-arc) was observed on the fringe bottom at 80 μs. The fringe pattern showed the change in density due to gas evolution or dissolution of the material. From the shape of the fringe pattern, this was attributed to the dissolution of material ejected from the anode. The delay of the light emission from the starting of the ejection was attributed to the micro-arc ignition in the interior of the anode. From 120 to 320 μs, the ejected material expanded upward, and a bubble at the ejection point became large. The fringe pattern is extended to the horizontal direction and upward. The fully formed bubble by the first micro-arc detached from the anode without a burst at 400 μs. The bubble was spherical in shape, 76 μm in diameter at 440 μs. The second micro-arc was observed from 120 to 360 μs adjacent to the first micro-arc. The second micro-arc appeared to grow the second bubble on the right side. If the discharge time of each micro-arc is less than 40 μs, as shown in Figure 11, this is the effect of several micro-arcs or a cascade discharge. The bubble burst around the micro-arcs could not be observed during the observation. Therefore, it was difficult to backfill the discharge pores with the ejected materials during the PEO process for Ti. In the case of the PEO process for Al, the ejected material from the anode was pressed onto the anode surface by the bubble burst around a micro-arc [12], which fabricated dense and flattened discharge craters, resembling thermal-sprayed splats [26]. Hence, the behavior of the micro-arcs and bubbles determines the structure of the PEO coating.

### 3.4. Coating Growth Behavior during PEO Process

The coating thickness during a conventional anode oxidation process can be evaluated from the current density and treatment time by Faraday’s law [27,28]. Although this applies to the PEO process [29,30], it is necessary to evaluate the variation of the input current density during the process. Therefore, it is better to evaluate the coating thickness from the total charge density passing through the anode during the PEO process. The increase in coating thickness was evaluated by the total charge density and can be expressed as

(1) $$\mathrm{dx}=\frac{M}{n\rho F}\mathrm{Idt}=\frac{M}{n\rho F}\mathrm{dQ}$$

where x is the coating thickness, M is the average molecular weight of the oxide layer, n is the valence number, ρ is the density of oxide layer, F is the Faraday constant, I is the anode current density, t is the treatment time, and Q is the total charge density that passed through the anode. 

**Figure 12:**
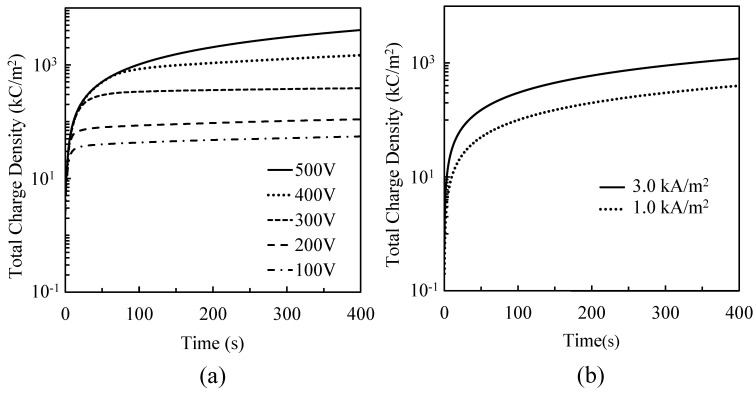
Time variation of the total charge density during the PEO process by (**a**) CV operation and (**b**) CC operation.

Figure 12a shows the time variation of the total charge density during the PEO process by CV operation during a 400 s treatment time. The total charge density was saturated with time when the applied voltage was ≤400 V by CV operation. The saturated point coincided with the time the micro-arcs were extinguished, as shown in Figure 5a. Hence, coating growth stops after the saturated point. The total charge density and coating thickness increased with voltage at 60 s of treatment time, as shown in Table 2. Figure 12b shows the time variation of the total charge density during the PEO process by CC operation during the 400 s treatment time. The total charge density was evaluated by the input current density. Thus, the total charge density increases linearly with the input current density. The maximum charge density was three times the difference because of the input current density.

During the PEO process for titanium, the formation of titanium oxide on the anode surface can be expressed as [13]

(2) $$\mathrm{Ti}+2H_{2}O\to {\mathrm{TiO}}_{2}+4H^{+}+4e^{-}$$

This reaction formula determines the valence number n as 4 in Equation (1). If the oxide layer is composed of an anatase phase of TiO_2_, the density of the oxide layer ρ is assumed to be 3830 kg/m^3^ [25]. Although the theoretical oxide coating thickness was estimated from the total charge density during the PEO process using Equation (1), as shown in Figure 12, this does not consider the evolution of gaseous bubbles on the anode surface, as shown in Figure 7 and Figure 11. The reaction of gas evolution is expressed as [28]

(3) $$2H_{2}O\to O_{2}\left(g\right)+4H^{+}+4e^{-}\;$$

In the conventional anodization process of Ti, the evolution of gaseous oxygen consumed 85% of the input current density and increased linearly with the input current density [28]. Hence, no more than 15% of the total charge density was utilized for oxide layer formation. Here, the utilization efficiency of the total charge density η is expressed as follows:

(4) $$\eta =\frac{Q_{\mathrm{oxide}}}{Q_{\mathrm{th}}}\times 100=\frac{x_{\mathrm{oxide}}}{x_{\mathrm{th}}}\times 100$$

where Q_oxide_ is the total charge density that contributes to the formation of an oxide layer, Q_th_ is the total charge density that passes through the anode during the treatment time, x_oxide_ is the oxide thickness, and x_th_ is the estimated theoretical oxide thickness by Q_th_. Except for the gas evolution, the internal discharge within the coating due to the porous structure of the PEO coating for Ti dissipates the charge density without increasing the coating thickness. The delay of the micro-arc from the material ejection in Figure 11 was attributed to the effect of internal discharge in the PEO coating [7]. The utilization efficiency of the total charge density for coating growth is essential for estimating coating thickness in theory.

**Figure 13:**
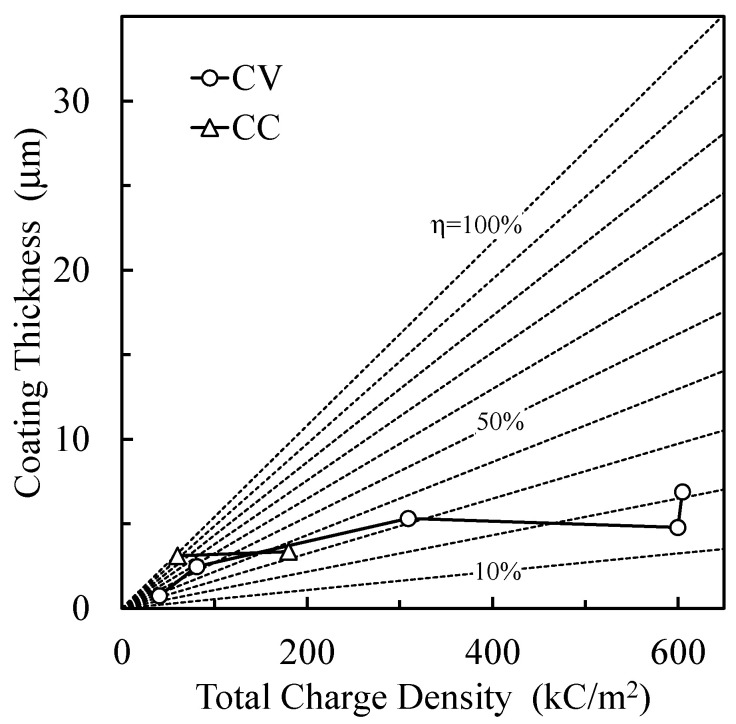
Coating thickness dependence on the total charge density by CV and CC operations and Faraday’s model with different utilization efficiency of total charge density.

Figure 13 shows the coating thickness dependence on the total charge density by CV operation and by CC operation during 60 s treatment time. The theoretical oxide thickness according to Faraday’s model with different utilization efficiencies of the total charge density is depicted as a dotted line in Figure 13. In CV operation, the coating thickness increased with the total charge density (applied voltage). The utilization efficiency of the total charge density decreased from 44.7% at 200 V to 16.0% at 500 V. In CC operation, the coating thickness slightly increased with the total charge density. The utilization efficiency of the total charge density decreased from 93.7% at 124 V to 31.4% at 193 V. Thus, the increase in voltage between the electrodes decreases the utilization efficiency of the total charge density. If the internal discharge within the coating increases as the voltage between the electrodes increases, it will improve the porous structure and mechanical strength of the oxide layer, as shown in Figure 5b. The utilization efficiency of the total charge for the PEO process by CV operation above 300 V was lower than that for the conventional anodization process. The utilization efficiency of the total charge density for the PEO process by CC operation was higher than that for the conventional anodization process. The decrease in utilization efficiency is attributed to the increase in gas evolution during the PEO process. The gas evolution induced the generation of the pore inside of the coating and the difference in the coating morphology between the CV (Figure 5b) and CC operations (Figure 6b). In CV operation at 400 V or higher, the pores inside of the coating partially reduced the resistivity of the coating [6] and induced the generation of micro-arcs inside of the coating after extinguishment of micro-arcs on the coating surface. These affected the discharge current, as shown in Figure 5a, and lowered the resistivity of the coating, as shown in Table 2. The gas evolutions by CC operation were lower than those by CV operation above 300 V. It is thought that the influence of the pores inside of the coating on the resistivity after extinguishment of micro-arc on the coating surface by CC operation was small in comparison with that by CV operation.

## 4. Conclusions

The behaviors of micro-arcs, bubbles, and coating growth by the PEO process for three titanium alloys were investigated to understand the coating formation process, and the following results were obtained.

(1) In constant voltage operation, the coating thickness increased with the applied voltage, and the maximum coating thickness of 6.88 μm by 60 s treatment time was obtained at 500 V. Although the surface roughness increased as the discharge voltage was increased, a low friction coefficient was achieved at 500 V. 

(2) In constant current operation, there was no apparent change in the morphology and thickness of the coating according to the current density. The roughness and friction coefficient decreased as the current density was increased.

(3) The PEO process, by short treatment operation, enabled an evaluation of individual micro-arcs and discharge craters on the anode. The minimum discharge diameter of the micro-arcs was 8 μm, and the discharge craters had a discharge pore of 0.3 μm in diameter in the center and petal-shaped burrs around the discharge pore. There were no bubble bursts around the micro-arcs and no backfilling of the discharge pores by the ejected materials.

(4) The coating thickness can be estimated from the total charge density and its utilization efficiency. The utilization efficiency of the total charge density decreased with increasing voltage between the electrodes. The utilization efficiency of the total charge for the PEO process by CV operation above 300 V was lower than that by the conventional anodization process. The utilization efficiency of the total charge for the PEO process by CC operation was higher than that by the conventional anodization process.

## Figures and Tables

**Table 1:** Operating conditions of the PEO process.

Operation	Applied Voltage (V)	Input Current Density (kA/m^2^)	Treatment Time (s)	Treatment Area (mm^2^)
Constant voltage (CV)	100–500	-	60, 400	50
Constant current (CC)	-	1.0, 3.0	60, 400	50
Short treatment time (ST)	Charge voltage 500	-	<0.1	1

**Table 2:** Thickness and resistivity of the PEO coating by CV operation.

Voltage (V)	Thickness (μm)	Resistivity (Ωm)
100	0.74	3.95 × 10^6^
200	2.47	1.09 × 10^6^
300	5.31	5.23 × 10^5^
400	4.78	4.14 × 10^4^
500	6.88	7.25 × 10^3^

**Table 3:** Resistivity of rutile TiO_2_ [25].

Temperature (K)	Resistivity (Ωm)
773	3.00 × 10^5^
1073	1.20 × 10^2^
1473	8.50 × 10^2^

**Table 4:** Thickness and resistivity of the PEO coating by CC operation.

Current (kA/m^2^)	Thickness (μm)	Resistivity (Ωm)
1.0	3.10	1.71 × 10^7^
3.0	3.36	1.66 × 10^7^

**Table 5:** Surface roughness and friction coefficient of Ti-15-3 and the PEO coating.

Material			Roughness (μm)	Friction Coefficient	
				No Lubrication	Lubrication
Ti-15-3	-		-	0.315	0.287
PEO coating	CV	200 V	0.3641	0.196	0.093
		500 V	0.9875	0.082	0.054
	CC	1.0 kA/m^2^	0.2642	0.197	0.092
		3.0 kA/m^2^	0.2591	0.144	0.070

## Data Availability

Not applicable.

## References

[1] Yerokhin A.L., Voevodim A.A., Lyubimov V.V., Zabinski J., Donley M. (1998). Plasma electrolytic fabrication of oxide ceramic surface layers for tribotechnical purposes on aluminium alloys. Surf. Coat. Technol..

[2] Simechen F., Sieber M., Kopp A., Lampke T. (2020). Introduction to plasma electrolytic oxidation-An overview of the process and applications. Coatings.

[3] Kaluđerović M.R., Schreckenbach J.P., Graf H.L. (2016). Titanium dental implant surfaces obtained by anodic spark deposition—From the past to the future. Mater. Sci. Eng. C.

[4] Aliofkhazraei M., Macdonald D.D., Matykina E., Parfenov E.V., Egorkin V.S., Curran J.A., Troughton S.C., Sinebryukhov S.L., Gnedenkov S.V., Lampke T. (2021). Review of plasma electrolytic oxidation of titanium substrates: Mechanism, properties, applications and limitations. App. Surf. Sci. Adv..

[5] Buling A., Zerrer J. (2019). Increasing the application fields of magnesium by ultraceramic^®^: Corrosion and wear protection by plasma electrolytical oxidation (PEO) of Mg alloys. Surf. Coat. Technol..

[6] Tsai D.S., Chou C.C. (2018). Review of the soft sparking issues in plasma electrolytic oxidation. Metals.

[7] Clyne T.W., Troughton S.C. (2019). A review of recent work on discharge characteristics during plasma electrolytic oxidation of various metals. Int. Mater. Rev..

[8] Yerokhin A.L., Snizhko L.O., Gurevina N.L., Leyland A., Pilkington A., Matthews A. (2003). Discharge characterization in plasma electrolytic oxidation of aluminium. J. Phys. D Appl. Phys..

[9] Yerokhin A.L., Snizhko L.O., Gurevina N.L., Leyland A., Pilkington A., Matthews A. (2004). Spatial characteristics of discharge phenomena in plasma electrolytic oxidation of aluminium alloy. Surf. Coat. Technol..

[10] Matykina E., Berkani A., Skeldon P., Thompson G.E. (2007). Real-time imaging of coating growth during plasma electrolytic oxidation of titanium. Electrochim. Acta.

[11] Jaspard-Mécuson F., Czerwiec T., Henrion G., Belmonte T., Dujardin L., Viola A., Beauvir J. (2007). Tailored aluminium oxide layers by bipolar current adjustment in the plasma electrolytic oxidation (PEO) process. Surf. Coat. Technol..

[12] Nishikawa R., Yasui T., Fukumoto M. Influence of micro-arc and bubble on film formation by plasma electrolytic oxidation. Proceedings of the 5th International Conference on Plasma Nanotechnology & Science (IC-PLANTS 2012).

[13] Stojadinović S., Vasilić R., Petković M., Kasalica B., Belča I., Žekić A., Zeković L. (2013). Characterization of the plasma electrolytic oxidation of titanium in sodium metasilicate. Appl. Surf. Sci..

[14] Nominé A., Troughton S.C., Nominé A.V., Henrion G., Clyne T.W. (2015). High speed video evidence for localised discharge cascades during plasma electrolytic oxidation. Surf. Coat. Technol..

[15] Troughton S.C., Clyne T.W. (2018). Cathodic discharges during high frequency plasma electrolytic oxidation. Surf. Coat. Technol..

[16] Kazek-Kęsik A., Krok-Borkowicz M., Pamuła E., Simka W. (2014). Electrochemical and biological characterization of coatings formed on Ti–15Mo alloy by plasma electrolytic oxidation. Mater. Sci. Eng. C.

[17] Wang Y., Yu H., Chen C., Zhao Z. (2015). Review of the biocompatibility of micro-arc oxidation coated titanium alloys. Mater. Des..

[18] Yerokhin A.L., Nie X., Leyland A., Matthews A. (2000). Characterisation of oxide films produced by plasma electrolytic oxidation of a Ti-6Al-4V alloy. Surf. Coat. Technol..

[19] Habazaki H., Onodera T., Fushimi K., Konno H., Toyotake K. (2007). Spark anodizing of β-Ti alloy for wear-resistant coating. Surf. Coat. Technol..

[20] Ceschini L., Lanzoni E., Martini C., Prandstraller D., Sambogna G. (2008). Comparison of dry sliding friction and wear of Ti6Al4V alloy treated by plasma electrolytic oxidation and PVD coating. Wear.

[21] Yao Z., Shen Q., Niu A., Hu B., Jiang Z. (2014). Preparation of high emissivity and low absorbance thermal control coatings on Ti alloys by plasma electrolytic oxidation. Surf. Coat. Technol..

[22] Nakajima M., Miura Y., Fushimi K., Habazaki H. (2009). Spark anodizing behaviour of titanium and its alloys in alkaline aluminate electrolyte. Corros. Sci..

[23] Tsunekawa S., Aoki Y., Habazaki H. (2011). Two-step plasma electrolytic oxidation of Ti-15V-3Al-3Cr-3Sn for wear-resistant and adhesive coating. Surf. Coat. Technol..

[24] Habazaki H., Tsunekawa S., Tsuji E., Nakayama T. (2012). Formation and characterization of wear-resistant PEO coatings formed on β-titanium alloy at different electrolyte temperatures. Appl. Surf. Sci..

[25] Diebold U. (2003). The surface science of titanium dioxide. Surf. Sci. Rep..

[26] Fukumoto M., Yamaguchi T., Yamada M., Yasui T. (2007). Splash splat to disk splat transition behavior in plasma-sprayed metallic materials. J. Thermal. Spray Technol..

[27] Albella J.M., Montero I., Sánchez O., Martínez-Duart J.M. (1986). Theoretical approach for the constant voltage stage in anodic oxidation. J. Electrochem. Soc..

[28] Hwang B.J., Hwang J.R. (1993). Kinetic model of anodic oxidation of titanium in sulphuric acid. J. Appl. Electrochem..

[29] Parfenov E.V., Yerokhin A., Matthews A. (2009). Small signal frequency response studies for plasma electrolytic oxidation. Surf. Coat. Technol..

[30] Mortazavi G., Jiang J., Meletis E.I. (2019). Investigation of the plasma electrolytic oxidation mechanism of titanium. Appl. Surf. Sci..

